# Experimental Study of Hysteresis behavior of Foam Generation in Porous Media

**DOI:** 10.1038/s41598-017-09589-0

**Published:** 2017-08-21

**Authors:** S. Kahrobaei, S. Vincent-Bonnieu, R. Farajzadeh

**Affiliations:** 1 Delft University of Technology, Delft, The Netherlands; 2 Shell Global Solutions International B.V., Rijswijk, The Netherlands

## Abstract

Foam can be used for gas mobility control in different subsurface applications. The success of foam-injection process depends on foam-generation and propagation rate inside the porous medium. In some cases, foam properties depend on the history of the flow or concentration of the surfactant, i.e., the hysteresis effect. Foam may show hysteresis behavior by exhibiting multiple states at the same injection conditions, where coarse-textured foam is converted into strong foam with fine texture at a critical injection velocity or pressure gradient. This study aims to investigate the effects of injection velocity and surfactant concentration on foam generation and hysteresis behavior as a function of foam quality. We find that the transition from coarse-foam to strong-foam (i.e., the minimum pressure gradient for foam generation) is almost independent of flowrate, surfactant concentration, and foam quality. Moreover, the hysteresis behavior in foam generation occurs only at high-quality regimes and when the pressure gradient is below a certain value regardless of the total flow rate and surfactant concentration. We also observe that the rheological behavior of foam is strongly dependent on liquid velocity.

## Introduction

Foam can greatly reduce gas mobility in porous media and therefore has been considered for a variety of environmental and industrial (subsurface) applications including aquifer remediation and improved-oil recovery^1–3^. The success of a foam-injection process primarily depends on the propagation of foam deep inside the porous medium, which is in turn influenced by the balance between the rates of the creation and destruction of foam lamellae. If the rate of the lamella creation exceeds that of the destruction foam is “generated” resulting in a significant drop in the gas mobility^4^. The magnitude of the gas mobility reduction is affected by foam texture, defined as the number of lamellae per unit volume. Fine-textured or strong foam encompasses a large bubble density and consequently imposes a large resistance against the gas flow. On the contrary, the bubble sizes are large in the coarse-textured foam resulting in a moderate modification of the gas mobility. Weak foam and coarse foam are often interchangeably used in literature; however, in this paper weak foam is referred to foams whose created lamellae are unstable; whereas, foam with limited number of created lamellae is referred to as coarse foam^4^. A considerable fraction of gas is trapped within the pores because of creation of the foam lamellae.

Foam generation in porous media is a complex function of many parameters including rock permeability, rock morphology, pressure gradient, flow velocity, surfactant type and concentration, salt type and concentration, saturation of the fluids, hysteresis, etc. The general consensus is that at least two perquisites must be satisfied for foam generation in porous media: (1) there should be sufficient amounts of a foaming agent in the aqueous phase, and (2) the pressure gradient (or velocity) should exceed a certain threshold^4–10^. The exact dependence of minimum pressure gradient,∇*p*_min_, on parameters such as permeability and foam quality (gas fractional flow, *f*_g_) is not yet well-established. For instance, according to Ransohoff and Radke^6^, ∇*p*_min_ scales linearly with *f*_g_; however, Rossen and Gauglitz’s^8^ experiments exhibit an opposite trend, which is attributed to the mobilization of a larger density of the liquid lenses promoting foam generation by the lamellae-division mechanism. For a fixed injection rate, the “jump” from coarse foam to strong foam occurs at lower injection rate if the liquid fraction of the injected fluids is increased^11^. In some cases, foam properties depend on the history of the flow or concentration of the surfactant, i.e., the hysteresis effect. In the percolation theory of Rossen and Gauglitz^8^ existence of the initial lenses and lamellae is necessary for foam generation by lamella division. Consequently, foam can show hysteresis behavior by exhibiting multiple states at the same injection conditions^12^. This is related to the history of foam generation, where coarse-textured foam is “abruptly” converted into strong foam with a fine texture at a critical injection velocity or pressure gradient^4, 9, 12^. Kibodeaux, *et al*.^13^ and Simjoo, *et al*.^14^ also observed a hysteresis behavior; however, they reported a more gradual shift from coarse to strong foam as the total velocity increased. Moreover, a shear-thickening behavior was observed at high gas volume fractions.

Foam rheology in porous media is strongly linked to its gas volume fraction. At higher gas fractional flows, i.e. in the so-called high-quality regime, the steady-state pressure gradient (measured along the porous medium) becomes nearly independent of the gas superficial velocity^11, 15^. In this regime, bubble size is very sensitive to injection rates and foam behavior is dominated by coalescence governed by the limiting capillary pressure^11, 16, 17^. In contrast at lower gas fractional flows or low-quality regime, the bubble size is presumably fixed at roughly pore size and does not change with the injection rate^15, 18^. Consequently, at this regime the pressure gradient is nearly independent of the liquid superficial velocity.

Despite its importance, the number of the published literature on the hysteresis behavior of foam generation in porous media is limited. Most of these studies have focused on the higher gas volume fractions (high-quality regime) and/or relatively high velocities, whereas foam can exhibit different rheological behavior at lower gas volume fractions (low-quality regime). It is therefore our objective to investigate the effects of injection velocity and surfactant concentration on foam generation and hysteresis behavior as a function of foam quality. This is achieved by performing series of core flooding experiments. In the first set of experiments, the total injection velocity is gradually varied, while the gas volume fraction is kept constant. In the second set of experiments, both the total injection velocity and the foam quality are kept constant, while the surfactant concentration is gradually varied.

The structure of the paper is as follows: First, the experimental methodology is described in Section 2. Section 3 provides the results of the so-called foam-quality-scan experiments^19, 20^ to distinguish the low- and the high-quality regimes. Furthermore, the details of the hysteresis in foam generation and its dependence on the total injection velocity and the surfactant concentration are discussed in Sections 3. We end the paper with some concluding remarks.

## Experiments

### Chemicals and Materials

#### Surfactant solution

Anionic Alpha Olefin Sulfonate (AOS) C_14–16_ (Stepan® BIO-TERGE AS-40 KSB) was used as the foamer; the properties of the foam films stabilized by this surfactant can be found in Farajzadeh, *et al*.^21^. The critical micelle concentration (CMC) of the surfactant in demineralized water with 1 wt% (~0.17 M) of NaCl was measured as 0.008 wt% (~6.27 × 10^−5^ M) using the Du Noüy ring method. In this study, the surfactant concentration for the flow-rate experiments was chosen as 0.5 wt% (~0.04 M) and for the surfactant-concentration experiments it varied between 0.008 wt% and 2 wt% (~0.15 M).

#### Gas

Nitrogen (N_2_) with a purity of 99.98% was used as the gas phase in our experiments.

#### Porous media

A 17-cm cylindrical quasi-homogenous Bentheimer sandstone core with a diameter of 3.8 cm was used as the porous medium. The average porosity of the core was 0.23. The permeability of the core was measured to be 2.3 × 10^−12^ m^2^ (±0.005 × 10^−12^ m^2^).

### Experimental Setup

**Figure 1:**
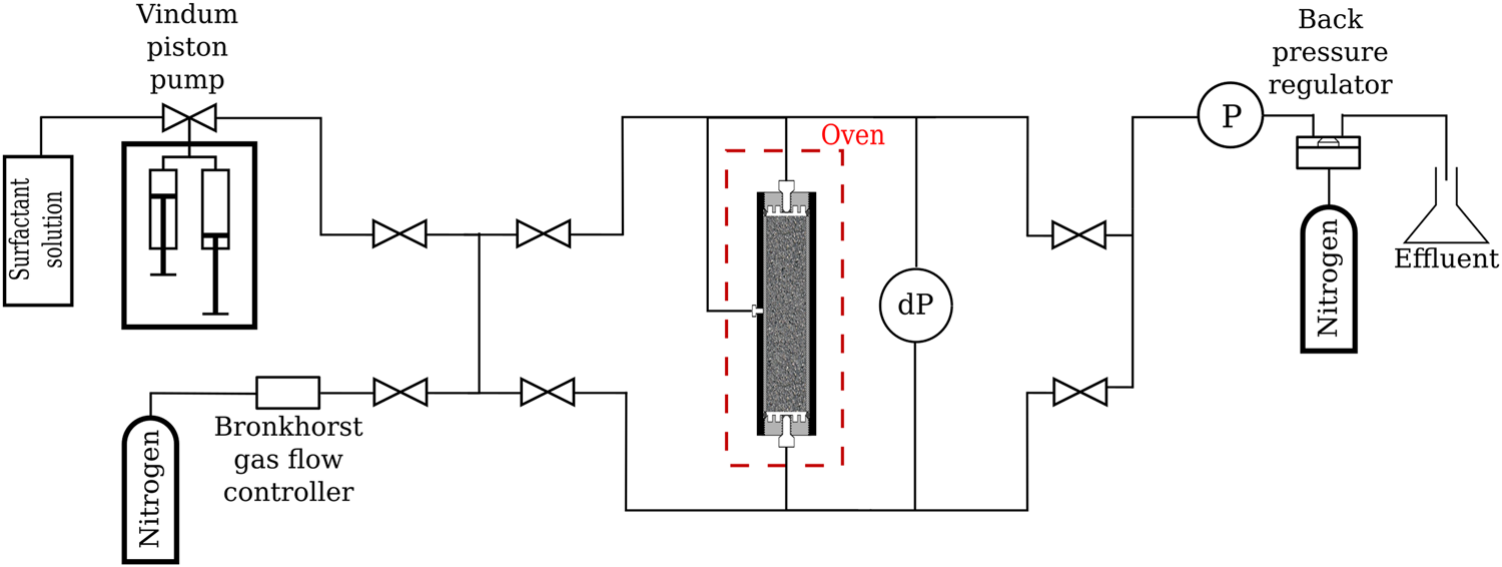
Schematic of the experimental apparatus.

The schematic of the experimental apparatus is shown in Fig. 1. The core sample was vertically placed inside a cylindrical core-holder. The core-holder was located inside an oven to keep the temperature constant at 30 °C. The overall pressure difference along the core was measured using a differential pressure transducer connected to the input and the output lines of the core-holder. The outlet of the core was connected to a back-pressure regulator (BPR) to maintain a constant pressure of 25 bar at the core outlet. The accuracy of the pressure transducers are 1 mbar. A calibrated Bronkhorst mass-flow controller was used to control the gas flowrate. Surfactant solution was injected at a constant rate using a Vindum double-effect piston-displacement pump. All the measurement instruments were connected to a data acquisition system, to record data with a frequency of 5 seconds.

It is well known that foam can be generated *in situ* either by continuous co-injection of gas and liquid or by injection of alternating slugs of surfactant solution and gas (SAG). Nevertheless, co-injection of gas and surfactant solution is a common practice in core-flooding experiments^4, 22–24^. Huh and Handy^24^ did a detailed comparison between SAG and simultaneous injection of gas and surfactant solution. However, it is important to note that simultaneous injection of gas and surfactant solution (foaming agent) could result in foam generation close to the core face inside the tubing. The continuous supply of foaming agent leads to the large number of lamellae and thus in greater mobility reduction compared to alternating injection of surfactant solution and gas^24, 25^. Foam texture is affected by lamellae creation, trapping, mobilization and destruction, which are highly dependent on the nature of the porous medium^24, 26^. Foam can exhibit a completely different behavior in bulk (i.e., tubing) compared to the porous media (i.e, core)^23^.

### Experimental Procedure

#### Core saturation and permeability measurement

After a leak test, 10 pore volumes of CO_2_ were injected into the core to remove the air from the core. Afterwards, brine was injected at an elevated pressure (25 bar) to dissolve and carry away the CO_2_. Subsequently, the core permeability was calculated by measuring the pressure drop along the core at different brine flow rates using Darcy’s law.

#### Surfactant injection

After permeability measurement, the core was pre-saturated with the surfactant solution to satisfy the rock adsorption of surfactant. Note that in the experiments with varying surfactant concentration, the core was pre-saturated with the surfactant solution at CMC concentration.

#### Foam-quality-scan experiments

Nitrogen was co-injected with the surfactant solution at different volume fractions into the core to generate foam. For all volume fractions, the gas phase and the surfactant solution were co-injected with a constant total flowrate of 1.0 ml/min corresponding to a total superficial velocity of 1.4 × 10^−5^ m/s. The co-injection was continued until the steady-state pressure was obtained for the respective fractional flow of gas.

#### Experiments with varying total injection flow rate

In this set of experiments, the total flow rate of gas and surfactant solution was varied in increasing steps while the gas volume fraction was kept constant. These experiments were begun from a low total flow rate. Once the steady-state pressure profile was obtained the flow rate was then increased to reach a new steady state. The total flow rate was increased in a stepwise manner to higher values until strong foam was generated and shear-thinning behavior was observed. Subsequently, the flow rate was reduced in steps down to the lowest flow rate. The difference between the steady-state pressures in the upward (increasing flow rate) and the downward directions (decreasing flow rate) is an indication of the hysteresis in generation of foam in porous media. The experiments were repeated for several gas volume fractions. After the completion of every experiment the core was cleaned, using a procedure explained below, and the permeability was measured to ensure similar initial condition for the next experiment. Table 1 provides a summary of the experiments in which total flow rate was varied.

**Table 1:** Summary of the experiments with varying total rates.

Experiment No.	Gas volume fraction [−]	Total flow rate range [ml/min]
1	0.40	0.17 to 1.20
2	0.70	0.10 to 0.80
3	0.80	0.15 to 1.40
4	0.90	0.10 to 2.50
5	0.95	0.10 to 1.80

#### Experiments with different surfactant concentrations

In these experiments, the surfactant concentration was gradually increased from the critical micelle concentration (CMC) to the higher surfactant concentrations and then it was decreased again to the CMC. The experiments were conducted with fixed total flow rate and gas volume fraction. Once again the difference between the measured steady-state pressures in the upward (increasing concentration) and the downward (decreasing concentration) directions reveals the effect of the surfactant concentration on the nature of the hysteresis behavior in foam flow. This set of experiments was also repeated with different gas volume fractions. After completion of every experiment the core was cleaned and prepared for the next experiment. Table 2 summerizes this set of experiments.

#### Core-cleaning procedure after foam experiments

After each test, the core was flushed by 10 pore volumes of an alcohol solution with 50 wt% of propanol to kill the foam. This was followed by 10 pore volumes of brine injection. Subsequently, the system pressure was reduced to the atmospheric pressure and increased again to 25 bar in order to remove the trapped foam. After that, the pressure was reduced to 1 bar and the core was vacuumed for several hours. The cleaning procedure was continued by injecting 10 pore volumes of CO_2_ followed by injecting 10 pore volumes of brine at 25 bar. Finally, the permeability of the core was measured to ensure that the initial state of the core has been restored.

## Results and Discussions

The measured steady-state pressure drops are converted into apparent viscosity using Darcy law,

1 $${\mu }_{app}=\frac{kA}{{q}_{g}+{q}_{l}}\,\frac{|{\rm{\Delta }}p|}{L},$$

where *k* [m^2^] is the absolute permeability, *A* [m^2^] is the cross-sectional area, *q*_*g*_ [m^3^/s] and *q*_*l*_ [m^3^/s] represent the flowrates of the gas phase and the surfactant solution (liquid phase), respectively, and Δ*p* [Pa] is the pressure drop along the core length, *L* [m]. Moreover, the fractional flow of the gas phase or foam quality (*f*_*g*_) is defined by,

2 $${f}_{g}=\frac{{q}_{g}}{{q}_{g}+{q}_{l}}.$$

The error bars for each data point were calculated using the standard deviation of the steady-state-pressure measurements.

### Foam-quality-scan experiment

**Figure 2:**
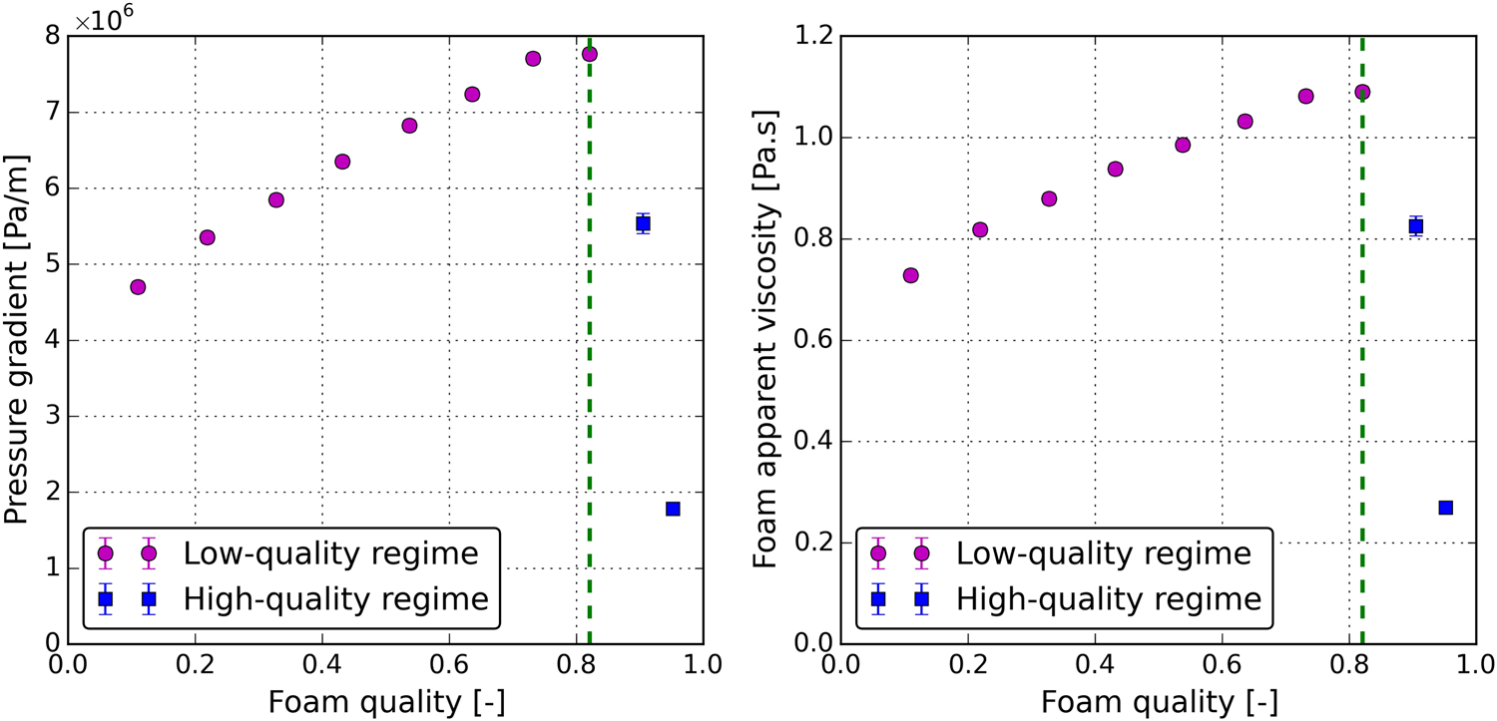
(**a**) The pressure gradient along the core and (**b**) the calculated apparent viscosity at different fractional flows of nitrogen. The total flowrate was set to a constant value of 1 ml/min. Purple circles and blue squares show the low- and high-quality regimes, respectively. The vertical green dashed line separates the low- and the high-quality regimes.

Prior to the hysteresis experiments, a foam-quality-scan was conducted to determine the foam quality at which the transition from high-quality to low-quality regime occurs. This is referred to as the *transition foam quality, f*_*g*_^*tr*^. The measured pressure drops and the calculated apparent viscosities for different foam qualities are shown in Fig. 2a and b, respectively. The foam apparent viscosity increases with the increase in the foam quality (low-quality regime) until it reaches a maximum at the transition foam quality (*f*_*g*_^*tr*^) and decreases afterwards (high-quality regime). The vertical green dashed line in Fig. 2 separates the low- and high-quality regimes. For the system investigated in this study, the transition foam quality is determined to be 0.8 and the maximum apparent viscosity is calculated to be around 1.1 Pa.s.

### Effect of total velocity on foam generation hysteresis

**Figure 3:**
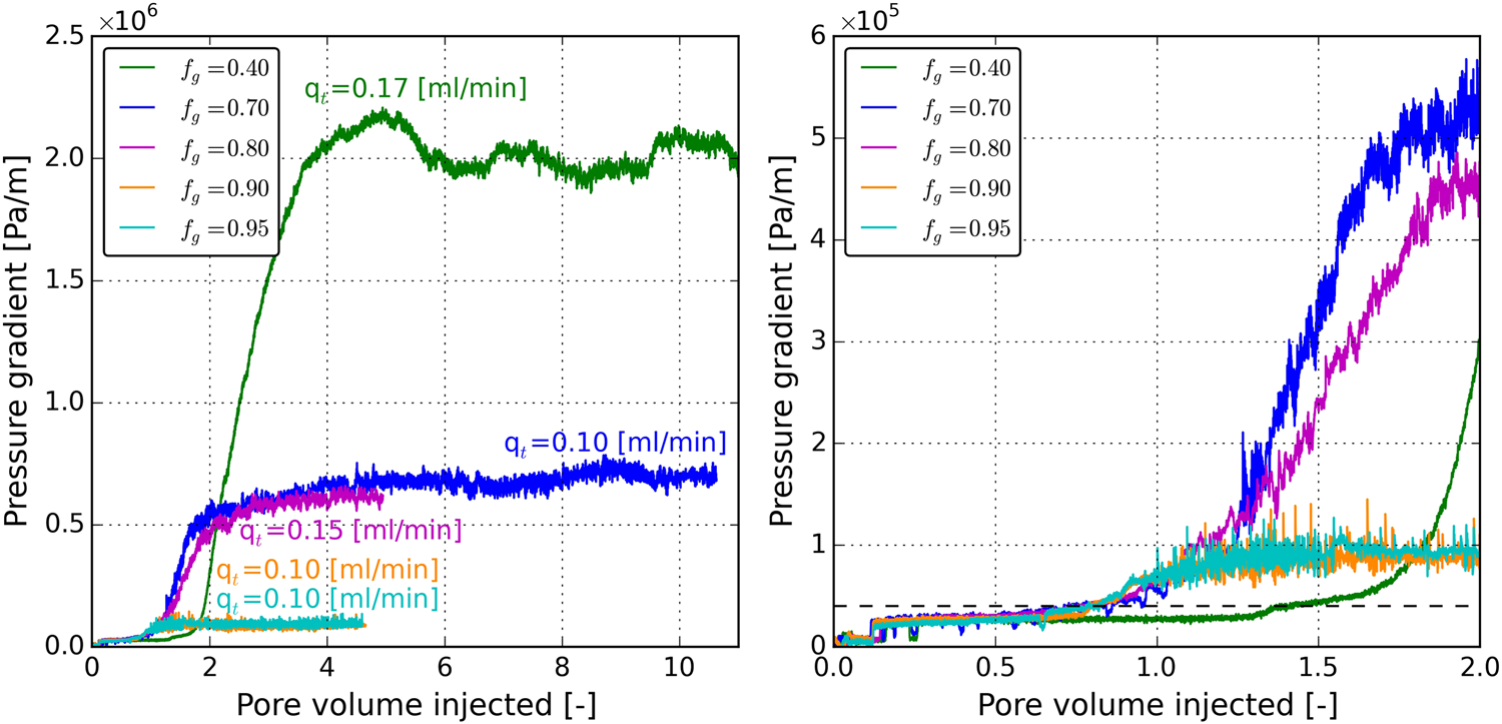
(**a**) The measured pressure history of the first step of the experiments, i.e., the lowest injection rate (see Table 1) for different foam qualities. (**b**) Magnification the first two pore volumes of the injection. The horizontal black dashed line in Fig. 3b indicates the critical pressure gradient in which the transition from coarse foam to strong foam occurs.

Five different foam qualities, summarized in Table 1, were chosen at low- and high-quality regimes to investigate the effect of flow rate or velocity on the hysteresis behavior of foam generation in porous media. This study uses the measured steady-state-pressure data to compare the foam strength (or apparent viscosity) for the different cases tested, especially when hysteresis in foam generation is discussed. However, before turning into the steady-state data we examine the dynamic behavior of foam generation in porous media. Figure 3a shows the measured pressure history of the first step of the experiments, i.e., the lowest injection rate (see Table 1) for different foam qualities. Figure 3b magnifies the first two pore volumes of the injection. In all experiments, the pressure starts to increase upon simultaneous injection of the gas and the surfactant solution. During the first pore volume (PV) of the injection the pressure remains low indicating that the created foam has a coarse texture. Notably, once the pressure gradient rises to the value of 0.4 × 10^5^ Pa/m (1.7 psi/ft) the transition from coarse foam to strong foam occurs in all experiments regardless of the foam quality or the total injection velocity. The horizontal black dashed line in Fig. 3b indicates the critical pressure gradient in which the transition from coarse foam to strong foam occurs. For the high-quality experiments, the transition appears to be sudden; however, for the low-quality experiments the transition is more gradual. Indeed, for the experiments in the low-quality regime it takes more than one pore volume of injection (and in the case of *f*_*g*_ = 0.4 more than 2PV) to reach the second steady state or strong foam state. Recall that at this stage of the experiments, the flowrates are fixed; therefore, it can be inferred from our data that it is possible to generate very strong foams (with apparent viscosity as high as 1200 cP) at very low velocities (as low as 1.41 × 10^−6^ − 2.46 × 10^−6^ m/s Darcy velocity). Our experiments also confirm the existence of a minimum pressure gradient rather than a minimum velocity for foam generation.

**Figure 4:**
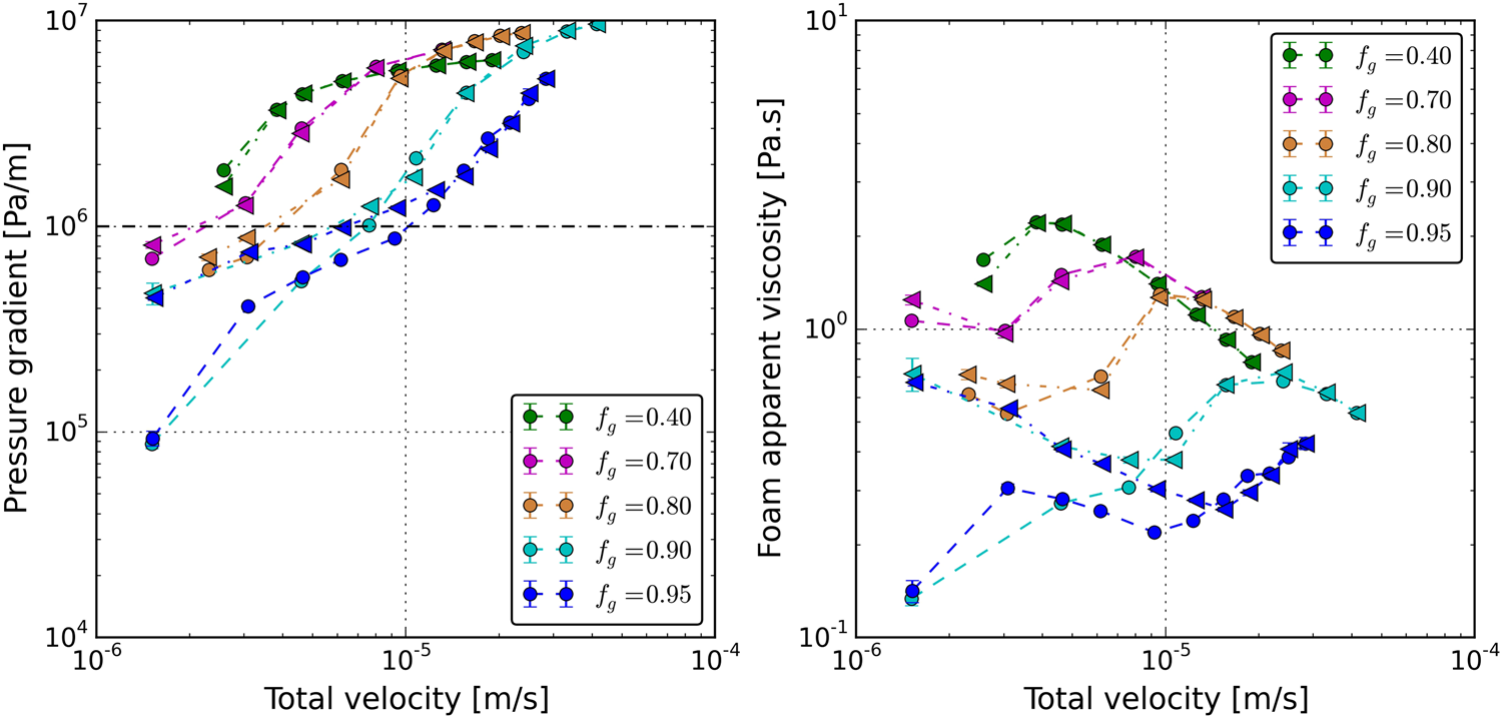
(**a**) The pressure gradient along the core and (**b**) the calculated apparent viscosity at different total velocities. The horizontal dashed line in 4a indicates the critical pressure gradient below which the hysteresis in foam generation occurs.

Figure 4a shows the pressure gradient along the core as a function of the total injection velocity. The calculated apparent viscosities are illustrated in Fig. 4b. Circles represent the measured data in the upward direction (increasing velocity) and the triangles represent the measurements in the downward direction (decreasing velocity).

The increase of pressure in Fig. 4 is an indication of creation of foam in the core. The calculated apparent viscosities are all above 0.120 Pa.s (~120 cP), which once again confirms that for all velocities strong foams are generated. For a fixed foam quality the pressure gradient increases with the increasing total velocity. The hysteresis behavior is only observed for the experiments conducted in the high-quality regime, i.e., for high-quality experiments it is difficult to generate foam when the experiment is begun with a low velocity. However, once foam is generated at higher velocities, foam will remain stable even when the velocity drops to smaller values. It can be seen that in our experiments, the hysteresis in foam generation occurs when the steady-state pressure gradient is below 1 × 10^6^ pa/m. This critical pressure gradient is indicated by a horizontal dashed line in Fig. 4a.

Because all steady-state foams are already strong, we do not observe a transition from coarse to strong foam regime. However, it is possible to detect a gradual transition from *relatively* strong to *very* strong foam for all foam qualities. For example this transition occurs at total velocities of 1 × 10^−5^ m/s and 2 × 10^−6^ m/s for foam qualities of 95% and 40%, respectively. The pressure gradients corresponding to this intermediate transition increase with decreasing foam quality, which could be attributed to the availability of more liquid lenses in low quality foam to generate more lamellae by lamella-division mechanism.

**Figure 5:**
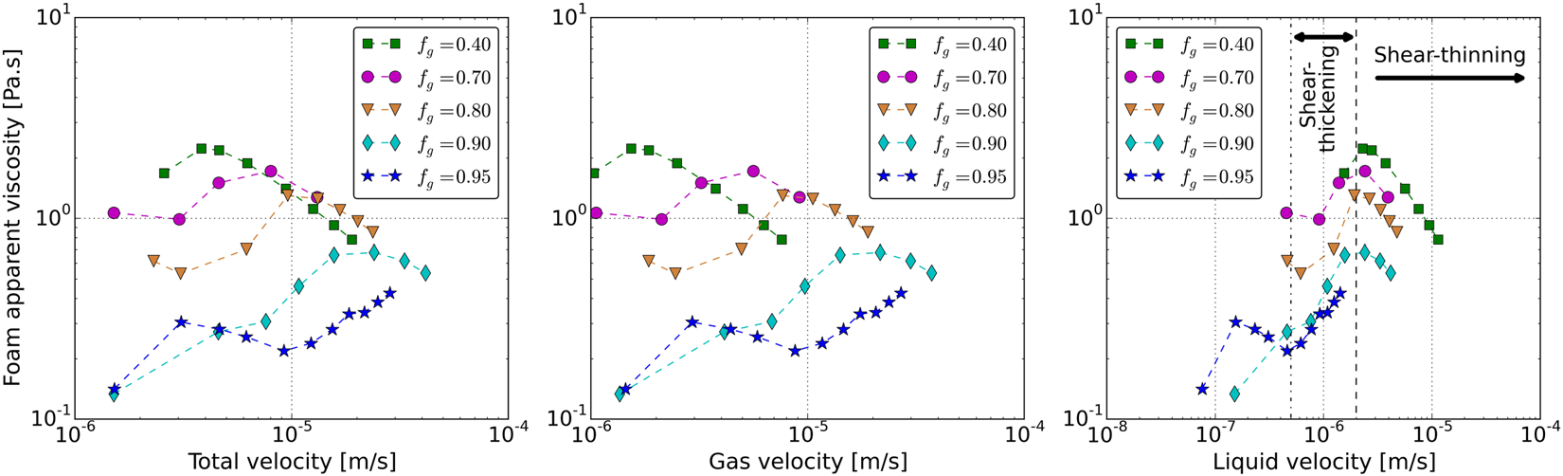
The calculated apparent viscosity as a function of (**a**) total velocity, (**b**) gas velocity and (**c**) liquid velocity. The vertical black dashed line in Fig. 5c indicates the minimum liquid velocity above which the generated foams show a shear-thinning behavior. Moreover, the vertical black dot-dashed line and the vertical black dashed line in Fig. 5c indicate the liquid velocity range in which the generated foams show a shear-thickening behavior.

Figure 4a also reveals that coarser foams are generated at higher foam qualities for all velocities examined. Three distinguished rheological regimes are observed for foam in porous media as a function of total velocity, as shown in Fig. 4b. For a fixed foam quality, at lower total velocities foam exhibits a shear-thinning behavior, until an intermediate total velocity at which the behavior becomes shear thickening. By further increase of the total velocity the foam apparent viscosity increases. At higher total velocities the foam becomes shear thinning again. The total velocity at which the behavior switches from the shear-thinning to the shear-thickening behavior increases with the increasing foam quality. To further investigate this phenomenon, the calculated apparent-viscosity data are plotted in Fig. 5 as functions of the total velocity (5a), the gas velocity (5b), and the liquid velocity (5c). The variations of the foam apparent viscosity are strongly correlated with the liquid velocity at different foam qualities. Figure 5c depicts that the switch between the two rheological behaviors (from shear-thinning to shear thickening and vice versa) occurs at a fixed liquid velocity for all gas volume fractions examined. Indeed the shift from initial shear-thinning region to the shear-thickening region occurs at the liquid velocity 0.5 × 10^−6^ m/s. The switch from the shear-thickening to the shear thinning behavior takes place at the liquid velocity of 2.0 × 10^−6^ m/s. The vertical black dashed line in Fig. 5c indicates the minimum liquid velocity above which the generated foams show a shear-thinning behavior. Moreover, the vertical black dot-dashed line and the vertical black dashed line in Fig. 5c indicate the liquid velocity range in which the generated foams show a shear-thickening behavior.

**Figure 6:**
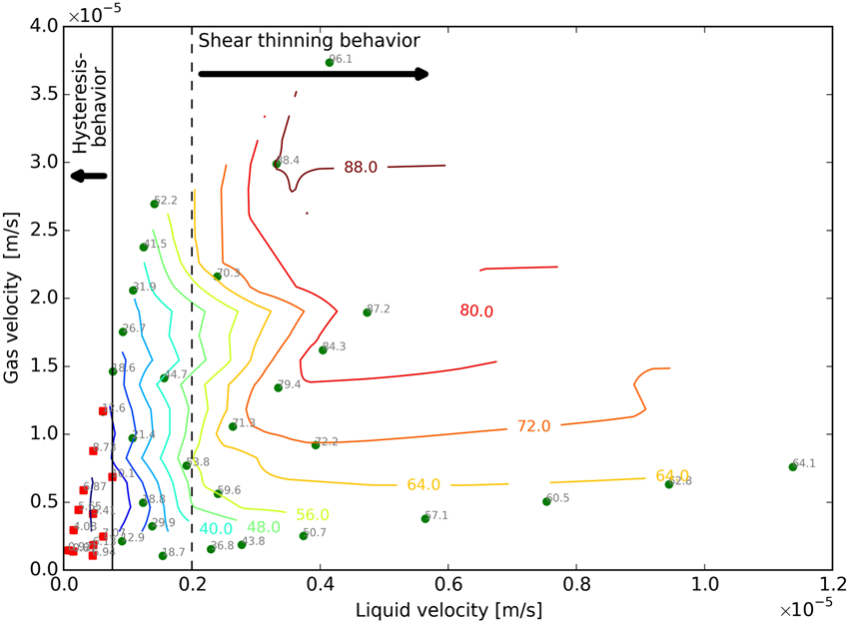
The steady-state pressure gradient in pa/m for coinjection of 0.5 wt% AOS 14–16 and nitrogen into a Bentheimer sandstone core. The circle and square markers represent the measurements, and the solid lines are the contour plots, obtained by the 2D spline interpolation. The red squares depict the points for which the hysteresis behavior in foam generation was observed. The vertical black solid line indicates the liquid velocity below which the foam generation exhibits hysteresis behavior. The vertical dashed line indicates a minimum liquid velocity above which the foam has a shear-thining behavior.

Figure 6 shows the steady-state pressure-gradient map obtained from the flow-rate experiments. The circle and square markers represent the measurements and the solid lines are the contour plots, obtained by the 2D-spline interpolation. The red squares depict the points for which the hysteresis in foam generation is observed. The vertical black solid line represents the maximum liquid velocity below which the hysteresis in foam generation was observed. The vertical black dashed line indicates the minimum liquid velocity above which the generated foams show a shear-thinning behavior. In general, in our experiments the foam-generation hysteresis is observed only for high-quality regime, where the pressure gradient is below 1 × 10^6^ pa/m. This pressure gradient corresponds to the liquid velocities below 7.6 × 10^−7^ m/s, which is indicated by the vertical solid line in Fig. 6.

### Effect of surfactant concentration on foam generation hysteresis

Five different surfactant concentrations, summarized in Table 2, were used at low- and high-quality regimes to investigate the dependency of the foam generation and its hysteresis on the variations in the surfactant concentration. Because the magnitude of the limiting capillary pressure increases with the increasing surfactant concentration, the transition foam quality is expected to decrease with the reduction in the surfactant concentration.

**Figure 7:**
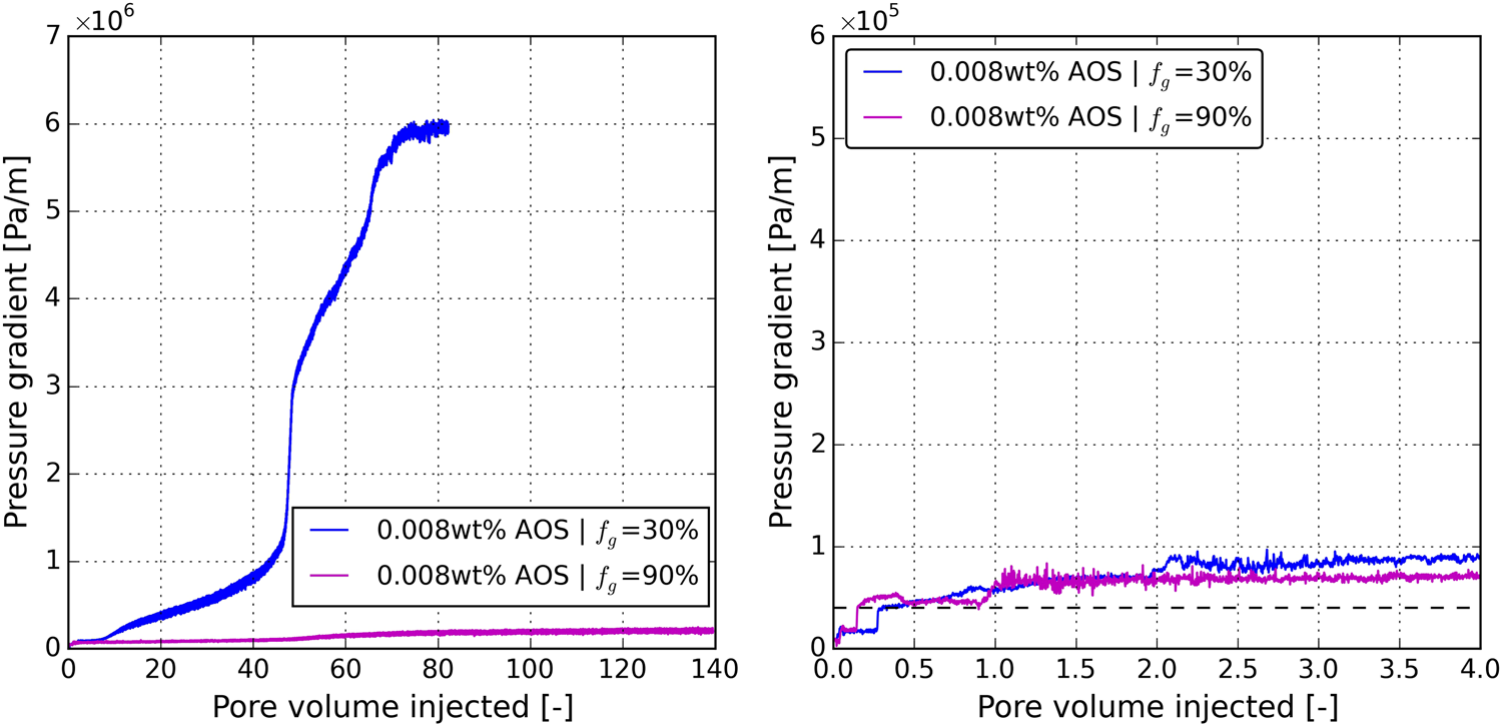
(**a**) The measured pressure history of the first step of the surfactant-concentration experiments, i.e., the lowest surfactant concentration (see Table 2) for different foam qualities. (**b**) Magnification the first four pore volumes of the injection. The horizontal black dashed line in Fig. 7b indicates the critical pressure gradient in which the pressure gradient starts to build up.

Figure 7a shows the measured pressure history of the first step of the hysteresis experiments, i.e., the lowest surfactant concentration (see Table 2) for different foam qualities. Figure 7b magnifies the first four pore volumes of the injection. At higher foam quality (*f*_g_ = 0.90) the pressure gradient starts to build up from 0.4 × 10^5^ Pa/m and increases to 2.05 × 10^5^ Pa/m (~*μ*_app_ = 0.0311 Pa.s) after 140 pore volume of injection. At lower foam quality (*f*_g_ = 0.30), the pressure gradient starts to build up slowly from 0.4 × 10^5^ Pa/m and increases to 5.9 × 10^6^ Pa/m (~ *μ*_app_ = 0.8 Pa.s) after 80 pore volume of injection. The horizontal black dashed line in Fig. 7b indicates the critical pressure gradient in which the pressure gradient starts to build up.

**Table 2:** Summary of the surfactant-variation experiments.

Experiment No.	Gas volume fraction [−]	Surfactant concentration range [wt%]
1	0.30	0.008% to 2%
2	0.90	0.008% to 1.5%

**Figure 8:**
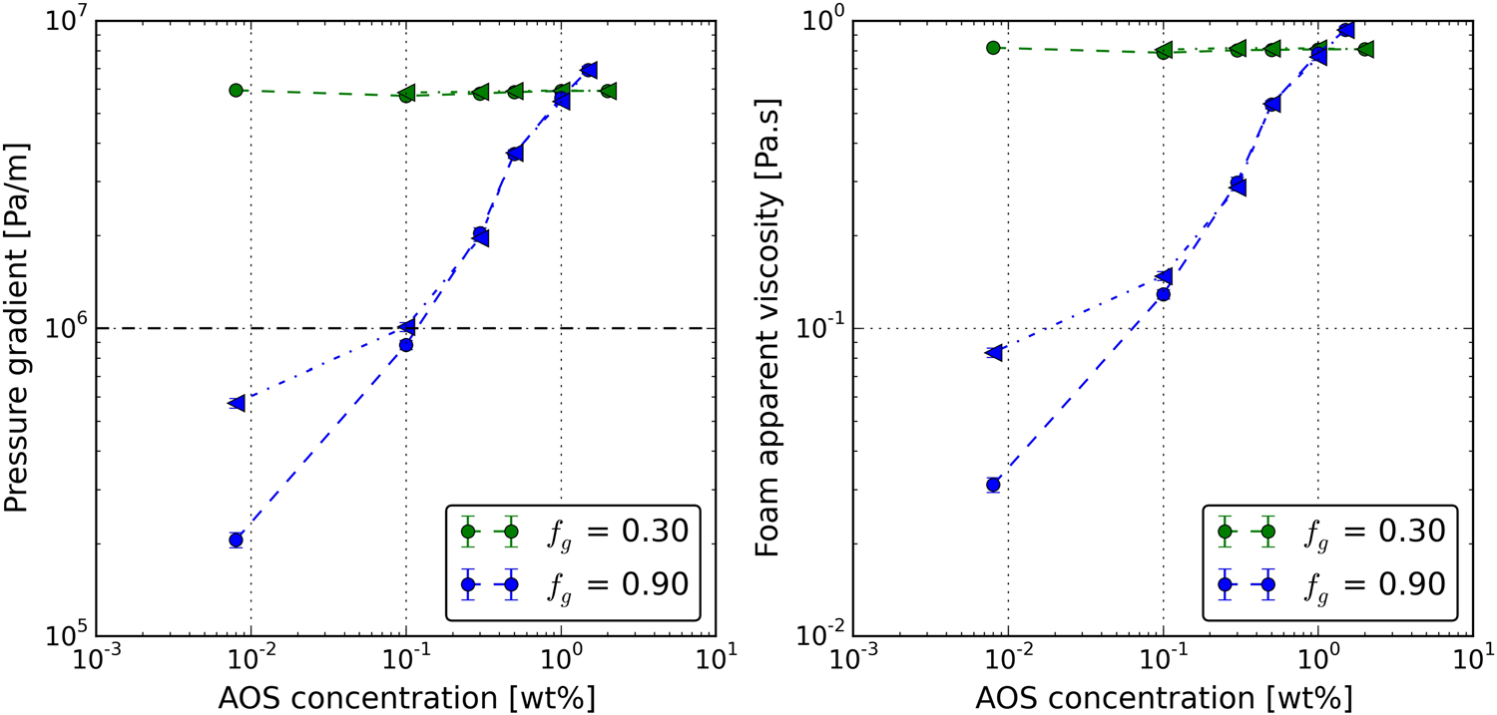
(**a**) The pressure gradient along the core and (**b**) the calculated apparent viscosity at different surfactant concentrations. The horizontal black dashed line in 8a indicates the critical pressure gradient below which the hysteresis in foam generation occurs.

Figure 8a shows the measured pressure gradient and Fig. 8b shows the calculated apparent viscosity as a function of surfactant concentration for the two examined foam qualities. The circles indicate the data in the upward direction (increasing concentration) and the triangles represent the data in the downward direction (decreasing concentration). The foam quality of 0.30 (green points) lies in the low quality regime for all surfactant concentrations and the foam quality of 0.90 (blue points) is in the high-quality regime (see Eftekhari, *et al*.^27^ and Eftekhari and Farajzadeh^28^).

The steady-state pressure gradient along the core or the calculated apparent viscosity is not impacted by the surfactant concentration in the low-quality regime, as shown in Fig. 7. The data in the upward and downward concentration follow the same path. Therefore, the steady-state pressure profile of the foam remains unchanged by the changes in the surfactant concentration and no hysteresis behavior is observed. On the other hand, in the high-quality regime (*f*_*g*_ = 0.90) the pressure gradient across the core and the apparent viscosity both increase as the surfactant concentration increases. This is because in the high-quality regime, foam stability is tuned by the limiting capillary pressure whose value increases with the increasing surfactant concentration^17^ and therefore foam becomes more stable as the surfactant concentration rises. In the downward direction, as the surfactant concentration decreases, the steady-state pressures deviate from those in the upward direction at a concentration of 0.1 wt%. This implies that foam stability is only slightly dependent on the surfactant concentration for concentrations above 0.1 wt%. Furthermore at the CMC concentration (0.008 wt%) the pressure gradient is increased by 178% from 2.0 × 10^5^ Pa/m to 5.7 × 10^5^ Pa/m. The foam apparent viscosity increases from 0.031 Pa.s 0.083 Pa.s.

Similar to the flow rate experiments, the hysteresis in foam generation is only observed in the experiments conducted in the high-quality regime for which the pressure gradient is below 1 × 10^6^ Pa/m. This critical pressure gradient is indicated by a horizontal black dashed line in Fig. 8a.

## Conclusions

In this study, the effect of changes in the total flow rate and the surfactant concentration on foam generation in porous media and its hysteresis behavior was investigated. To this end, several core flood experiments were conducted, in which the nitrogen gas and an AOS 14–16 surfactant solution were simultaneously injected into a Bentheimer sandstone core. For the cases investigated and under our experimental conditions, the following conclusions are made:

- At all flowrates examined in this study, corresponding to the superficial velocities of 1.46 × 10^−6^ to 3.67 × 10^−5^ m/s, very strong foams with apparent viscosities of larger than 0.133 Pa.s (~133 cP) were generated.
- The transition from coarse-foam to strong-foam regime appears to be independent of flowrate, surfactant concentration, and foam quality (~0.4 × 10^5^ Pa/m or 1.7 psi/ft).
- The hysteresis in foam generation only occurs in the high-quality regime, where the limiting capillary pressure governs the stability of foam.
- Generally, the hysteresis in foam generation in porous media occurs when the measured steady-state pressure gradient is below a certain value independent of flowrate or surfactant concentration. In our experiments the value of this critical pressure gradient was ~1 × 10^6^ Pa/m. This pressure gradient corresponds to the liquid velocities below 7.6 × 10^−7^ m/s.
- At very low and high velocities foam is shear-thinning, i.e., its apparent viscosity decreases as the flowrate increases. However, at intermediate velocities the behavior is shear thickening. The shear-thickening behavior occurred for the liquid velocities between 0.5 × 10^−6^ m/s and 2.0 × 10^−6^ m/s in all experiments.
- There is a minimum liquid velocity above which foams generated in both low- and high-quality regimes exhibit a shear-thinning behavior. The foams generated above this minimum liquid velocity are typically very strong.
- The changes in the surfactant concentration have significant influence on the steady-state pressure behavior of the foam at the high-quality regime because of the dependence of the limiting capillary pressure on the surfactant concentration.
